# Supplementary material for: A molecularly defined subpopulation of oligodendrocyte precursor cells controls the generation of myelinating oligodendrocytes during postnatal development
Source: PLoS Biol. 2024 Jul 10;22(7):e3002655. doi: 10.1371/journal.pbio.3002655 (PMC11236193; doi:10.1371/journal.pbio.3002655)
Supplement: Attachment — Submitted filename: OPC Paper - Response to Reviewers_V5.pdf [file pbio.3002655.s011.pdf]

Reviewer #1: This manuscript by Moghimyfiroozabad and colleagues presents c1ql1 as a novel marker for a subset of oligodendrocyte precursor cells (OPCs) that are crucial for myelination. They show that c1ql1 is expressed by approximately 50% of OPCs and that this population does not segregate to specific OPC clusters in single cell RNA sequencing data, nor does it overlap with established subgroup markers of committed or early differentiating cells. Ablation of c1ql1-expressing OPCs using an oligodendrocyte lineage specific DTA model results in a substantial lack of myelination despite the presence of other OPCs. Therefore, the authors conclude that c1ql1 defines a non-redundant OPC population that is crucial for the generation of myelinating oligodendrocytes, as stated in the title.

Work over the past 5 years or so has shown that oligodendrocyte precursors are not a homogenous population. They can exist side by side in different states as they progress along their lineage, and they can form functionally different groups depending on the local microenvironment that they reside in. Despite these experimental findings, there are still large gaps in our understanding on the roles and the identity of OPCs, largely because of the lack of markers that allow to distinguish OPC populations apart from markers of differentiation.

The present manuscript provides a novel molecular marker which in itself will be of great interest and benefit to the glial community. Moreover, it is a striking result to show how the loss of some OPCs leads to a near-complete absence of myelin. Although there are of course a lot of questions remaining as to how and why these phenotypes occur, and what the function of c1ql1 in OPCs might be, I regard the novelty and interest of the present data as they are as very high.

We are very grateful for this encouraging and positive assessment of our work, as well as for the suggestions of the reviewer. We have added new experiments and manuscript modifications in response to these suggestions, as described in our point-by-point answer below, and they have very much improved our manuscript.

I only have one major comment which I think should be addressed with additional data:

1. The statement that the remaining OPCs cannot compensate the loss of the c1ql1-pos OPCs needs to be substantiated with additional analysis because this statement is not valid from a single timepoint analysis at P16. The authors come to this conclusion because they refer to earlier work from the Richardson lab which has shown that ablation of all forebrain OPCs is compensated by a similar timepoint through repopulation by midbrain-derived OPCs. However, one needs to bear in mind that the timing of the ablation is different. In the Richardson work,

OPCs were eliminated from the very beginning (immediately after their specification in embryo and around birth), meaning that there was more time for compensation than in the experiments carried out here.

We agree with the reviewer and have now added additional experimental data to answer this point. These data show that the loss of the *C1ql1*^{pos} OPCs, starting around birth in our ablation model in the cortex, cannot be compensated by *Sox10* derived lineage even in two months old animals. In these animals, non-*Sox10* OPCs somewhat compensate in terms of OPC generation, as suggested by reviewer #2 and seen previously when OPCs are ablated in the adult (Cell Reports Meth. 2023 Feb 28;3(2):100414). However, and very strikingly, this does not enable functional compensation, since myelination is still extremely decreased in the brains of adult *C1ql1*-expressing OPCs ablated animals and almost completely absent in their cortex.

What the authors observe looks to me more like a timing effect with OPCs dying later, and limited by the circumstance that the animals do not survive much beyond P16. There are multiple ways to gain more insight into the capacity of compensation (or its lack of) and I cannot tell which one will be feasible for the authors. For example, how long does it take for OPCs to die from the moment they begin to express c1ql1 in the DTA ablation model. If death is not instantaneously, there would be even less time for OPC compensation.

As stated earlier, our analysis of adult *C1ql1*-expressing OPCs ablated animals shows that *Sox10* lineage derived OPCs cannot compensate for the ablation of *C1ql1*^{pos} OPCs. Only non-*Sox10* lineage derived OPCs are able with time to compensate for this loss, but cannot compensate for the deficits in terms of oligodendrocyte generation and myelination. The manuscript has been modified accordingly.

This would explain why there is no OPC reduction in the cortex but in the cc at P7 whereas there is some in the cc; presumably c1ql1 cells are present in the cc earlier than in the Ctx.
What should be feasible though is a more careful analysis of when c1ql1-expressing OPCs arrive in these different CNS regions, the timing of their disappearance, and analysis of the proliferative responses of the c1ql1-negative OPCs at different timepoints before P16. This would help understand the presented findings better.

We agree with the reviewer that knowledge of the time course of *C1ql1* expression during postnatal development in OPCs from the different CNS regions is important for better

understanding of our findings. To this aim we performed several experiments. First, we performed gene expression analysis using smFISH on P1 brains. These experiments showed some, but rare, *C1ql1*/*Cspg4* double positive cells in the cortex, suggesting that *C1ql1* starts to be expressed in a few OPCs in this region by birth (Fig S3B). However, quantification was made very difficult at this age, because of the density of nuclei and difficulties in proper segmentation. In a second set of experiments, we thus used a genetic strategy to assess the time course of expression in different regions more quantitatively at this early stage (Fig S3C). Using a cross between *C1ql1*^{Cre} and YFP reporter mice and immunolabeling with PDGRa and NG2 markers, we were able to quantify the percentage of OPCs expressing *C1ql1* at P1-P2 in the cortex and different parts of the corpus callosum. The data show that *C1ql1* expression arises in OPCs earlier in the corpus callosum than in the cortex. Approximately 10% of OPCs (213 / 2,133 cells) are YFP positive in the cortex, while about 40% (238 / 519 and 624 / 1,714 cells) are found in the corpus callosum at the same age. This is in line with the fact that effects of ablation of *C1ql1* expressing OPCs in the DTA model are already obvious in the corpus callosum by P7 while they are not visible in the cortex.

We completed our assessment of the timing of the disappearance of OPCs in the ablation model by adding a P0 timepoint (Fig S4A). The results show no change in the density of *Sox10*-GFP cells in any region analyzed and only a small decrease in the density of PDGFRa^{pos} *Sox10*-GFP cells, corresponding to OPCs in the lateral part of the center of the corpus callosum. This shows that the ablation is more precocious in the corpus callosum than in the cortex in accordance with the earlier expression of *C1ql1* in OPCs from this region (Fig S3C).

We also analyzed the proliferative responses of the *C1ql1* negative OPCs by immunolabeling for Ki67, a classic marker for proliferation (J Cell Physiol. 2000 Mar;182(3):311-22.), in the cortex of P0 and P7 *C1ql1*Cre/*Sox10*-DTA mice to determine whether increased proliferation in this region might delay OPC loss. The results show that the level of proliferation of oligolineage cells is very low in the cortex regardless of the genotype. No significant increase is detected after ablation of *C1ql1* positive OPCs (Fig S5). This can be explained in two ways. First ablation of *C1ql1* positive OPCs does not result in increased proliferative of *C1ql1* negative OPCs. A second hypothesis, more likely, is that *C1ql1* expression might be induced in OPCs before they engage in the proliferating cycle for compensation, and thus OPCs are killed before they divide. We have added a discussion of this point on page 12.

It would also very informative to learn about OPC numbers and potential compensation in animals that do survive longer that P16, even if n numbers will be low.

We managed to collect brains from three adult mice (range P71-P255). Analysis of these brains show that the loss of the *C1ql1* positive OPCs cannot be compensated by *Sox10* derived lineage even months after ablation started (Fig S6). In these animals, non *Sox10* lineage derived OPCs somewhat compensate in terms of OPC generation, as has been seen previously when OPCs are ablated in the adult (Cell Reports Meth. 2023 Feb 28;3(2):100414). However, and very strikingly, this does not enable functional compensation since myelination is still extremely decreased in the brains of these animals and almost completely absent in their cortex (Fig S9). Interestingly non-*Sox10* derived OPCs can generate oligodendrocytes in the center of the corpus callosum, but not in the lateral part and in the cortex (Fig 3). Consequently, myelination remains extremely reduced in the lateral part of the corpus callosum and completely absent in the cortex in adult mice after ablation of *C1ql1*-OPCs.

Because if c1ql1-OPCs cannot get compensated, it would mean that they are entirely distinct from the remaining OPCs, which is highly unlikely given that these cells can be found across different OPC clusters. It is probably more likely that any OPC can, in principle, become c1ql1 positive, depending on yet unknown cues (this would also be a worthy discussion point(!)).

We actually think, like the reviewer, that the likeliest explanation for our findings is that *C1ql1* expression is induced at a specific stage of OPC differentiation. This is supported by the fact that only non-*Sox10* lineage derived OPCs can finally compensate for *C1ql1*-OPCs ablation after several weeks. Interestingly our results would suggest that *C1ql1* induction happens when OPCs are induced to divide and differentiate into oligodendrocytes. Given our GO term analysis, the fact that non *Sox10* lineage derived OPCs cannot compensate for the generation of myelinating oligodendrocytes, and the lack of visible increase in proliferation in remaining OPCs, we think that *C1ql1* might be a marker of the specific transition described by Marisca et al. (2020). In this paper, the authors propose that certain OPCs can be induced to divide to specifically produce daughter cells that will differentiate into myelinating oligodendrocytes. We have now added this point in the discussion (page 12).

Minor points:

2. could the authors discuss why c1c1l /sox-DTA mice die so early, which is unlikely due to the lack of some OPCs and myelination reported here? Do they know if there are other cell types ablated outside the CNS?

C1ql1 is expressed by neurons and glia of the enteric nervous system (http://mousebrain.org/adolescent/genesearch.html and table below). Some of these cells and their precursors also express *Sox10* (cf. table below and https://www.nature.com/articles/pr200196). It is thus possible that the enteric nervous system of *C1ql1*/*Sox10*-DTA mice is affected. Mutations leading to enteric nervous system dysfunction and lethality in the first few months of life have been described (https://www.nature.com/articles/s41598-021-82785-1 and https://www.ncbi.nlm.nih.gov/pmc/articles/PMC6261281/). A sentence has been added to the text to suggest this point page 5.

Index	Name	Description	*C1ql1*	*Sox10*
183	ENT2	Nitrergic enteric neurons	6.56	0.635
184	ENT3	Nitrergic enteric neurons	5.46	0.535
185	ENT4	Cholinergic enteric neurons	0.465	1.07
215	COP1	Committed oligodendrocytes cells (COP)	0.607	1.08
239	OPC	Oligodendrocytes precursor cells	2.92	0.541
243	ENTG1	Enteric glia, proliferating	0.506	2.47
247	ENTG5	Enteric glia	0.445	1.62
248	ENTG6	Enteric glia	0.445	1.43
249	ENTG7	Enteric glia	0.577	1.23

3. it would help the reader if the authors could provide rough numbers/fractions of effect sizes throughout the results section. For example, at the beginning of the results section. What is the proportion of c1ql1 pos cells which are NeuN-negative? This would help the reader to get a better idea of significance of these observation.

We thank the reviewer for this suggestion. We have added rough numbers/ fractions of effect sizes wherever possible. Regarding the proportion of *C1ql1*(GFP)-pos NeuN-neg cells, the proportion would be very different from one region to another, depending on cell density (and the types of the neuronal cells present), and was not quantified here.

4. out of curiosity, a recent study of human white matter snRNA seq has also described two OPC subpopulations (Seeker et al., 2023; https://doi.org/10.1186/s40478-023-01568-z). Is it possible to tell how do the OPC_A and OPC_B populations seen in this other study compare to the OPC_1 and OPC_2 populations described here?

Six markers were described for the OPC_A and OPC_B populations (3 each) in the study that the reviewer is referring to. Only Sema3D was detected in a significant amount of cells in the Marques et al. 2018 dataset. Analysis of the distribution of those cells (Cf. UMAP plot on the right) does not show a preferential presence in one of the OPC clusters we have identified, thus suggesting that OPC_A and OPC_B do not correspond to our clusters.

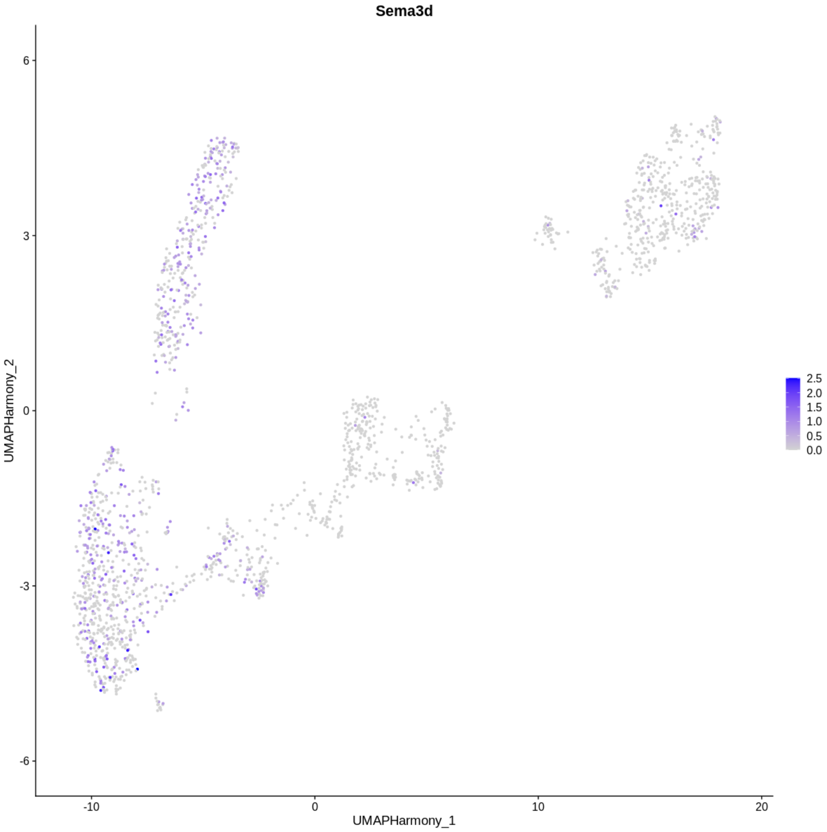

5. for better readability, it would be great if the authors could consider reworking their figures:

harmonise font sizes used, use headings within the figure panels so that one can grasp quicker what is being assessed here.

Thank you for this comment. Figures were all reworked based on the PLOS Biology guidelines and font sizes are harmonized. Headings are now added to the panels of the figures.

Reviewer #2: This paper investigates the extent to which genetic ablation of oligodendroglia defined by the expression of Sox10 and C1ql1 perturbs homeostatic control of the OPCs during postnatal development, and their capacity to generate oligodendrocytes. They present evidence that C1ql1 is expressed in a subpopulation of OPCs that appear to be poised to differentiate into oligodendrocytes and that ablating these C1ql1+ oligodendroglia during postnatal development results in OPC depletion and a marked reduction in oligodendrocyte density and myelination. Whilst the paper demonstrates that C1ql1 expression is likely an essential pre-requisite for the generation of oligodendrocytes during postnatal myelination, due to the nature of the genetic manipulation, it does not provide any insight into how C1ql1 is acting in OPCs or whether C1ql1 expression is required for postnatal myelination.

The authors demonstrate by crossing a C1ql1Cre knockin mouse line with the Cre-dependent R26Cas9-GFP line, that, in addition to neuronal populations, a subpopulation of NG2+ OPCs express GFP, indicative of Cre-mediated recombination. Additional evidence supporting the expression of C1ql1 by OPCs in wildtype mice is provided by duplex smFISH analysis which reveals that approximately 50-60% of NG2+ cells co-express C1ql1. The authors reanalyze published RNA-seq data that define the transcriptome of oligodendroglia in juvenile and adult mice to corroborate their expression data. Next, by crossing the C1ql1Cre line with the Cre-dependent Sox10DTA mouse line, the authors observed a ~75% and ~30% reduction in OPC density in the corpus callosum and cerebral cortex respectively, due to the induction of DTA expression in C1ql1-expressing OPCs. Intriguingly, whilst C1ql1 mRNA is present in ~70% of cortical OPCs at P7 and P15, a reduction in OPC density was not observed at P7, and was only reduced by 30% at P16. By contrast, OPC density in the corpus callosum is already diminished at P7 and the degree of this reduction more closely reflects the proportion of OPCs in this compartment that expresses C1ql1.

We are very grateful for the suggestions of the reviewer. Please find below our point-by-point response.

1.

The authors claim that this discrepancy reflects a selective failure of compensation for OPC loss in the corpus callosum. An alternate explanation is that C1ql1 expression in the corpus callosum precedes that in the cortex, and that recombination of the Sox10DTA allele occurs later in the cortex, meaning that DTA expression in OPCs in this region is delayed relative to the corpus callosum. To support their argument for differences in compensatory mechanisms, the authors

should provide evidence that cortical OPCs in C1ql1Cre; Sox10DTA mice are indeed undergoing apoptosis at P7.

Thank you for this comment. We realized that our wording might have been misleading. We completely agree that a different timing of expression of *C1ql1* in OPCs in different regions (corpus callosum versus cortex) is one key parameter that explains the different time course in OPC decrease in the ablation model. We have added new data on the timing of expression of *C1ql1* in OPCs (Fig S3) that clearly show a more precocious expression in the corpus callosum than in the cortex. We have now modified the manuscript accordingly, in particular page 6.

2.

The authors also suggest that since OPC migration into the dorsal forebrain is complete by P10, compensation for OPC loss does not occur beyond this time-point. This conclusion is inconsistent with their own data showing lack of compensation for OPC loss in the corpus callosum at P7 and evidence from adult OPC ablation experiments showing that partial OPC ablation in both gray and white matter can be compensated by residual non-ablated OPCs (Nat Neurosci. 2013 Jun;16(6):668-76, Cell Reports Meth. 2023 Feb 28;3(2):100414).

To assess whether the lack of compensation for OPC loss that we observe at P7 and P16 after ablation of *C1ql1* positive OPCs is simply a delay and would nonetheless occur with time (cf. reviewer 1's remarks as well), we assessed OPCs' presence in adult animals several months after the start of OPC loss. We managed to collect brains from three adult mice (range P71-P255). Analysis of these brains show that the loss of the *C1ql1* positive OPCs cannot be compensated by *Sox10* derived lineage even months after ablation started (Fig S6). In these animals, non *Sox10* lineage derived OPCs somewhat compensate in terms of OPC generation, as suggested by the reviewer and reported previously when OPCs are ablated in the adult (Cell Reports Meth. 2023 Feb 28;3(2):100414)). However, and very strikingly, this does not enable functional compensation since myelination is still extremely decreased in the brains of these animals and almost completely absent in their cortex (Fig S9). Interestingly non-*Sox10* derived OPCs can generate oligodendrocytes in the center of the corpus callosum, but not in the lateral part and in the cortex (Fig 3). Consequently, myelination remains extremely reduced in the lateral part of the corpus callosum and completely absent in the cortex in adult mice after ablation of *C1ql1*-OPCs.

3.

Next, the authors perform Gene Ontology analysis of a previously published RNA-seq dataset which provides evidence that C1ql1 expression by OPCs may reflect a specific transition state of OPCs poised to differentiate into oligodendrocytes but declines at the point at which newly formed oligodendrocytes are specified. Indeed, since C1ql1Cre; Sox10DTA mice exhibit an even higher degree of CC1+ cell loss than might be expected from the proportion of OPCs that express C1ql1, this raises the possibility that C1ql1 is expressed at a necessary transition step during the differentiation of OPCs into oligodendrocytes. If C1ql1 is indeed a late marker of OPCs that are poised to differentiate, one might anticipate that this expression coincides with a flux in the expression of genes associated with cell division. It would therefore be valuable for the authors to explore whether transcripts for mitosis-associated proteins differ between C1ql1-negative and C1ql1-positive subpopulations
of OPCs. This is also of particular interest given the previous data that links C1QL1 to cell proliferation and tumorigenicity.

The scRNASeq analysis of the previously published dataset from MArques et al. shows the correlation of expression of *C1ql1* with only one gene that is related to cell cycling: a tumor suppressor gene called *Gos2*, which might control quiescence in hematopoietic stem cells. This point is discussed on page 12 of the manuscript.

4.

Whilst the examination of CC1+ cell density in the cortex and corpus callosum provides compelling evidence that oligodendrocyte density is markedly reduced in C1ql1Cre; Sox10DTA mice, the examination of the effects upon myelin formation is less conclusive. Their analysis is complicated in part by focusing on immunohistochemical analysis of MBP expression, a myelin protein that is expressed at a relatively early stage of oligodendrocyte differentiation. Their data show significant residual MBP expression in the corpus callosum which leaves it unclear how myelination per se is altered in this tract in C1ql1Cre; Sox10DTA mice. It would be more informative to assess myelination across the forebrain using the fluoromyelin stain (J Neurosci Methods. 2012 Aug 15;209(2):344-50) or by SCoRe imaging (Nat Med. 2014 Apr;20(4):443-9) which specifically identify areas of compact myelin. Since electron microscopy is the gold standard for myelin assessment, the authors should also provide
EM data of the corpus callosum and cerebral cortex. This would serve to corroborate the

presumed myelination defects in these areas and to provide independent data to support their contention that the 19% reduction in the thickness of fiber tracts in the lateral corpus callosum of C1ql1Cre; Sox10DTA mice is due to changes in axonal compaction rather than in a deficit of axons.

To better characterize the deficits in myelination in the brains of *C1ql1* OPCs ablated mice, we used several approaches: staining with an anti-CNP antibody (another early marker of myelination), staining for Fluoromyelin and SCoRe imaging to visualize compact myelin in sections from both adult and P16 animals. CNP staining showed similar results as MBP at both ages. At P16, the level of myelin was difficult to assess with Fluoromyelin and SCoRe even in controls, suggesting that at that age myelin is still immature and lacks compaction. However, staining in adult sections were clear and strikingly demonstrated the lack of myelin in the cortex and lateral parts of the corpus callosum even months after ablation of *C1ql1*-OPCs. Those results are now presented in Fig 4, S8 and S9.

Minor points:

On p5, the authors state that "C1ql1 is not expressed in progenitors of OPCs (Figure 1D). Thus, the expression of the Cre recombinase using the C1ql1Cre locus induces DTA expression and death of the Cre-expressing cells in the oligolineage without affecting C1ql1-expressing neurons." It would be more accurate to also state that the Sox10 promoter driving expression of DTA is only active in the oligodendroglial lineage.

Thank you for this precision. We added the following sentence “*Sox10* promoter is specifically active in all the oligodendroglial lineage” (Page 5, line 2) before this part of the text “In the absence of Cre, all the oligolineage cells, OPC, COPs and OLs, express GFP in the $C1ql1^{wt}$; $Sox10^{DTA}$ mouse line. *C1ql1* is not expressed in progenitors of OPCs (Fig 1D)”.

For Figure S1, it would be preferable to use arrowheads in all subparts of the figure not just the merge of all fluorescent channels to illustrate the expression pattern of GFP+ cells more clearly.

Thank you for your suggestion. The arrowheads are added to all subparts of Fig S1.
