# Supplementary material for: A molecularly defined subpopulation of oligodendrocyte precursor cells controls the generation of myelinating oligodendrocytes during postnatal development
Source: PLoS Biol. 2024 Jul 10;22(7):e3002655. doi: 10.1371/journal.pbio.3002655 (PMC11236193; doi:10.1371/journal.pbio.3002655)
Supplement: Attachment — Submitted filename: Moghimyfiroozabadetal - Response to Reviewers_2nd Revision.pdf [file pbio.3002655.s012.pdf]

Reviewer #1: The authors have fully addressed all my points from the previous review. No further comments. Congratulations on this interesting work.

We are very thankful for your positive evaluation of our revised work, and your previous suggestions, comments and corrections that improved the quality of this study.

Reviewer #2 (Tobias D. Merson): The authors have conducted additional experiments and analyses that have addressed most of the concerns that I raised in my previous review and the manuscript is much improved. However, I have one major concern with the authors' interpretation of some of their data and the terminologies they use to describe them. The authors posit (lines 7-13, p.8) that the emergence of Sox10-GFPneg PDGFRapos NG2pos cells in the adult C1ql1Cre; Sox10DTA mouse brain suggests their origin from neural progenitor cells within the ventricular-subventricular zone (V-SVZ), rather than from Sox10 lineage-derived OPCs. They refer to these Sox10-GFPneg PDGFRapos NG2pos as "non-Sox10 lineage derived OPCs". However, this conclusion presents several challenges. To bolster their claim, the authors reference a study by Xing et al. (Cell Rep. Methods 3, 100414, 2023) which details the pharmacogenetic ablation of OPCs using Pdgfra-CreERT2; Sox10DTA mice coupled with intracisternal infusion of the anti-mitotic AraC. In this study, Xing and team illustrate that PDGFRA+ cells regenerated in the cerebrum after OPC ablation originate from neural precursor cells born in the V-SVZ. However, these NPC-derived OPCs were demonstrated to express GFP, indicating that the transgenic Sox10 promoter is active in OPCs that derive from the V-SVZ. Moreover, in contrast with the authors' claim that all oligolineage cells in Sox10DTA mice express GFP (lines 3-4, 22-23, p.5), the original characterization of Sox10DTA mice (Kessaris et al., 2016) highlights that some 2% of OL lineage cells do not co-express GFP. Additionally, Xing et al. (2023) also report that some OPCs in adult Sox10DTA mice do not express GFP, despite expression of endogenous SOX10 protein. In fact, contrary to their claim that all OL lineage cells express GFP, the authors highlight the presence of GFPneg CC1pos cells in the center of the corpus callosum of C1ql1Cre; Sox10DTA (line 14, p.9). These observations demonstrate that the transgenic Sox10 promoter in Sox10DTA mice, which drives GFP expression in non-recombined cells and DTA following Cre-mediated recombination, isn't universally active in all OL lineage cells. Moreover, the authors' use of the term "non-Sox10 lineage derived OPCs" is problematic as they only show that these cells do not express GFP; they have not shown that they fail to express SOX10 protein, which would be necessary to support the use of this terminology. A more accurate term would be "GFPneg OPCs". The most plausible interpretation

of the authors' findings in adult C1ql1Cre; Sox10DTA mice is the selective expansion of OPC clones where the transgenic Sox10 promoter remains inactive. Consequently, these OPCs fail to express either GFP or DTA. Considering that NPC-derived OPCs display activity of the transgenic Sox10 promoter, it follows that any NPC-derived OPCs that are generated after OPC depletion in C1ql1Cre; Sox10DTA mice, would also be expected to undergo DTA-mediated apoptosis. The authors' observation that surviving adult C1ql1Cre; Sox10DTA mice exhibit marked deficiency in OLs and myelin, despite an expansion in the population of GFPneg OPCs, raises the intriguing possibility that these GFPneg OPCs could be defective in their differentiative capacity in this context. The results and discussion sections of the manuscript need to be revised extensively to address these issues regarding the interpretation of their data.

We are very grateful for the reviewer's comments. We have made modifications to the manuscript accordingly.

Abstract :

p.1 line 21-22 and Intro p.3 line 6-7: replaced "*Sox10*-GFP$^{neg}$ OPCs" by "While some OPCs escape *Sox10*-driven ablation"

Results and discussion:

1. We made corrections to the text and cited the original paper (Kessaris et al., 2006) to highlight that *Sox10* drives GFP expression in not all, but "the vast majority of the oligolineage cells" in the *Sox10*$^{DTA}$ line:

p.5 line 4-5 "The *Sox10* promoter is specifically active in all the oligodendroglial lineage and drives GFP expression in more than 98% of the oligolineage cells in the *Sox10*$^{DTA}$ line [12]. Accordingly, in the absence of Cre *(C1ql1*$^{wt}$*; Sox10*$^{DTA}$ mice), the vast majority of the oligolineage cells, OPCs, COPs and OLs, are GFP positive (Fig 2B)."

p. 5 "Since GFP labels **nearly** the entire oligolineage cells in the *Sox10*$^{DTA}$ mouse model[12],"

2. We have made corrections to the text to highlight the fact that OPC compensation happens in the adult via cells that do not express GFP (low or no *Sox10* promoter activity) and escaped DTA-ablation.

Title of the paragraph p7 changed to "Loss of *C1ql1*-expressing OPCs is compensated by cells escaping *Sox10*-driven ablation."

Conclusion of the results lines 11-16, p. 8 : "This means that a compensation of OPC loss can occur after many weeks, but from cells in which the promoter of *Sox10* has low or no activity ( less than 2% of the cells [12,28]) and thus escape *Sox10*$^{DTA}$-mediated ablation (*Sox10*-GFP$^{neg}$ OPCs) in the presence of Cre recombinase. These cells could be derived locally or from neural progenitor cells in the ventricular-subventricular zone, as observed several weeks after ablation by pharmacological and genetic tools of all OPCs in the adult mouse brain[28]."

Discussion p. 11 line 8-9 : "With time, GFP$^{neg}$ OPCs in which the activity of the promoter of *Sox10* is likely very low or absent, repopulate the forebrain."

p. 11, lines 21-24 : "It has been reported that 4% of oligodendroglia in the corpus callosum are *Sox10*-negative[38]. A low percentage of *Sox10* expressing cells do not express GFP in *Sox10*$^{DTA}$ animals[12,28], due to low levels of activity of the *Sox10* promoter. Both types of cells could give rise to GFP$^{neg}$ OPCs in the adult *C1ql1*$^{Cre}$*; Sox10*$^{DTA}$ forebrain."

3. The term "non-*Sox10* lineage derived OPCs" is replaced by "GFP$^{neg}$ OPCs" in the manuscript (line 20, p. 9; line 12, p. 10; lines 26, 27, 29, p. 11) as well as the legends of Fig 3 (line 18, p. 25) and of Fig S6 (lines 21 & 26, p. 28).
